# β-Adrenergic Inhibition Prevents Action Potential and Calcium Handling Changes during Regional Myocardial Ischemia

**DOI:** 10.3389/fphys.2017.00630

**Published:** 2017-08-28

**Authors:** Shannon R. Murphy, Lianguo Wang, Zhen Wang, Philip Domondon, Di Lang, Beth A. Habecker, Rachel C. Myles, Crystal M. Ripplinger

**Affiliations:** ^1^Department of Pharmacology, University of California, Davis Davis, CA, United States; ^2^Department of Biomedical Engineering, University of California, Davis Davis, CA, United States; ^3^Department of Physiology and Pharmacology, Oregon Health & Science University Portland, OR, United States; ^4^Institute of Cardiovascular and Medical Sciences, University of Glasgow Glasgow, United Kingdom

**Keywords:** ischemia, arrhythmia, beta blocker, sarcoplasmic reticulum, calcium

## Abstract

β-adrenergic receptor (β-AR) blockers may be administered during acute myocardial infarction (MI), as they reduce energy demand through negative chronotropic and inotropic effects and prevent ischemia-induced arrhythmogenesis. However, the direct effects of β-AR blockers on ventricular electrophysiology and intracellular Ca^2+^ handling during ischemia remain unknown. Using optical mapping of transmembrane potential (with RH237) and sarcoplasmic reticulum (SR) Ca^2+^ (with the low-affinity indicator Fluo-5N AM), the effects of 15 min of regional ischemia were assessed in isolated rabbit hearts (*n* = 19). The impact of β-AR inhibition on isolated hearts was assessed by pre-treatment with 100 nM propranolol (Prop) prior to ischemia (*n* = 7). To control for chronotropy and inotropy, hearts were continuously paced at 3.3 Hz and contraction was inhibited with 20 μM blebbistatin. Untreated ischemic hearts displayed prototypical shortening of action potential duration (APD_80_) in the ischemic zone (IZ) compared to the non-ischemic zone (NI) at 10 and 15 min ischemia, whereas APD shortening was prevented with Prop. Untreated ischemic hearts also displayed significant changes in SR Ca^2+^ handling in the IZ, including prolongation of SR Ca^2+^ reuptake and SR Ca^2+^ alternans, which were prevented with Prop pre-treatment. At 5 min ischemia, Prop pre-treated hearts also showed larger SR Ca^2+^ release amplitude in the IZ compared to untreated hearts. These results suggest that even when controlling for chronotropic and inotropic effects, β-AR inhibition has a favorable effect during acute regional ischemia via direct effects on APD and Ca^2+^ handling.

## Introduction

Ventricular arrhythmias during the acute phase of myocardial infarction (MI) remain a leading cause of death (Henriques et al., 2005; Benjamin et al., 2017). Experimental studies indicate that ventricular arrhythmias peak between ~10 and 30 min of myocardial ischemia (Curtis, 1998; de Groot and Coronel, 2004; Di Diego and Antzelevitch, 2011). Ischemia-induced arrhythmias are multi-factorial and can be attributed to several changes in myocyte electrophysiology, including intracellular Ca^2+^ overload, extracellular K^+^ accumulation, slow conduction, shortening of the action potential duration (APD), increased dispersion of repolarization, and post-repolarization refractoriness (Akar and Akar, 2007; Di Diego and Antzelevitch, 2011; Coronel et al., 2012).

β-adrenergic receptor (β-AR) blockers have been shown to reduce ventricular arrhythmias during the acute phase of MI (Norris et al., 1984). β-AR blockers also limit infarct size, relieve pain, and reduce early mortality when administered during acute MI (López-Sendón et al., 2004). Recent evidence suggests that when administered prior to primary percutaneous coronary intervention (PCI), intravenous β-AR blocker therapy results in improved ejection fraction and fewer major adverse cardiac events compared to PCI without β-AR blocker pre-treatment (Halkin et al., 2004; Pizarro et al., 2014). The mechanisms underlying these improved outcomes are likely multi-factorial, but may stem in part from a global reduction in oxygen demand due to reductions in heart rate and contractility, as well as inhibition of β-AR signaling in the ischemic region.

Indeed, β-AR activation may be locally elevated in the ischemic region due to release of catecholamines from the ischemic cardiac sympathetic nerves (Schömig et al., 1984, 1987; Lameris et al., 2000; Killingsworth et al., 2004). This local, non-exocytotic catecholamine release is in response to intracellular acidification of the neurons and is independent of central sympathetic drive (i.e., occurs *in vivo* as well as in *in vitro* isolated ischemic hearts) (Schömig et al., 1987). Some studies indicate that the local norepinephrine (NE) concentration in the extracellular space can rise to as much as 100- to 1,000-fold higher than normal plasma concentrations within 10–30 min of no-flow ischemia or anoxia (Schömig et al., 1987; Kurz et al., 1995; Lameris et al., 2000). Significant elevation of interstitial NE has also been observed following several minutes of fibrillation-induced global ischemia (Killingsworth et al., 2004). Although less pronounced, myocardial interstitial levels of dopamine (DA, precursor to NE) and epinephrine (Epi) also rise significantly during ischemia (Schömig et al., 1984; Lameris et al., 2000). Thus, locally enhanced β-AR stimulation may exacerbate the arrhythmogenic effects of ischemia by contributing to Ca^2+^ overload and additional APD shortening due to β-AR augmentation of repolarizing K^+^ currents.

The goal of the present study was to determine the direct impact of β-AR inhibition on ventricular electrophysiology and sarcoplasmic reticulum (SR) Ca^2+^ handling in the ischemic myocardium. To accomplish this, we performed dual optical mapping of transmembrane potential (V_m_) and SR Ca^2+^ in isolated rabbit hearts. Using a low-affinity Ca^2+^ indicator (Fluo-5N AM, K_d_ ≈ 400 μM), free intra-SR Ca^2+^ can be directly monitored everywhere on the surface of the heart (Wang et al., 2014, 2015). Thus, SR Ca^2+^ release represents a rapid decrease in the signal (the inverse of the intracellular Ca^2+^ transient) and SR Ca^2+^ ATPase (SERCA) function can be directly assessed via the time constant of SR Ca^2+^ reuptake. Acute regional ischemia was induced by ligation of the left circumflex artery (LCA). To isolate electrophysiological effects from metabolic effects that may be secondary to negative chronotropy and inotropy induced by β-AR inhibition, hearts were continuously paced and contraction was abolished with the excitation-contraction uncoupler blebbistatin.

## Methods

### Ethical approval

All procedures involving animals were approved by the Animal Care and Use Committee of the University of California, Davis and adhered to the Guide for the Care and Use of Laboratory Animals published by the US National Institutes of Health (NIH Publication No. 85-23, revised 1996).

### Langendorff perfusion

Male New Zealand White rabbits (*n* = 19) weighing 3–3.5 kg were anesthetized with a single intravenous injection of pentobarbital sodium (50 mg/kg) containing 1000 IU heparin. Hearts were rapidly removed and perfused as previously described (Wang et al., 2014). Briefly, following cannulation of the aorta, Langendorff perfusion was initiated with oxygenated (95% O_2_, 5% CO_2_) modified Tyrode's solution of the following composition (in mmol/L): NaCl 128.2, CaCl_2_ 1.3, KCl 4.7, MgCl_2_ 1.05, NaH_2_PO_4_ 1.19, NaHCO_3_ 20, and glucose 11.1 (pH 7.4 ± 0.05). Flow rate (25–35 mL/min) was adjusted to maintain a perfusion pressure of 60–70 mmHg. Two Ag/AgCl disc electrodes were positioned in the bath to record an electrocardiogram (ECG) analogous to a lead I configuration. A bipolar pacing electrode was positioned on the base of the right ventricular epicardium for pacing, which was performed at a pacing cycle length (PCL) of 300 ms using a 2 ms pulse at twice the diastolic threshold.

### Dual optical mapping of SR Ca^2+^ and V_m_

Optical mapping of V_m_ and intra-SR free [Ca^2+^] ([Ca^2+^]_SR_) was performed as previously described (Wang et al., 2014, 2015). After stabilization of perfusion (~10 min), the excitation-contraction uncoupler blebbistatin (Tocris Bioscience, Ellisville, MO; 10–20 μM) was added to the perfusate. Hearts were then switched to a recirculating perfusate (200 mL) containing 5 μM Fluo-5N AM [Invitrogen, Carlsbad, CA; initially dissolved in 0.25 mL dimethyl sulfoxide (DMSO) and 0.25 mL 20% pluronic acid for a final concentration of 0.25 and 0.025%, respectively during the dye loading] for 60 min at room temperature, followed by a 15 min washout at 37°C. A lead I ECG was continuously monitored throughout the loading procedure. Hearts were subsequently stained with the voltage-sensitive dye RH237 (Invitrogen, Carlsbad, CA; 50 μl of 1 mg/ml in DMSO). All experiments were performed at 37°C.

The anterior epicardial surface was excited using LED light sources centered at 470 nm (Mightex, Pleasanton, CA) and bandpass filtered from 475 to 495 nm (Semrock, Rochester, NY). The emitted fluorescence was collected through a THT macroscope (SciMedia, Costa Mesa, CA) and split with a dichroic mirror at 593 nm (Semrock, Rochester, NY). The longer wavelength moiety, containing the V_m_ signal, was longpass filtered at 715 nm and the shorter wavelength moiety, containing the [Ca^2+^]_SR_ signal, was bandpass filtered with a 28 nm filter centered at 520 nm (Semrock, Rochester, NY). The emitted fluorescence signals were recorded using two CMOS cameras (MiCam Ultima-L, SciMedia, Costa Mesa, CA) with a sampling rate of 0.5–1 k Hz, and 100 × 100 pixels with a 31 × 31 mm field of view.

### Experimental protocol

**Figure 1:**
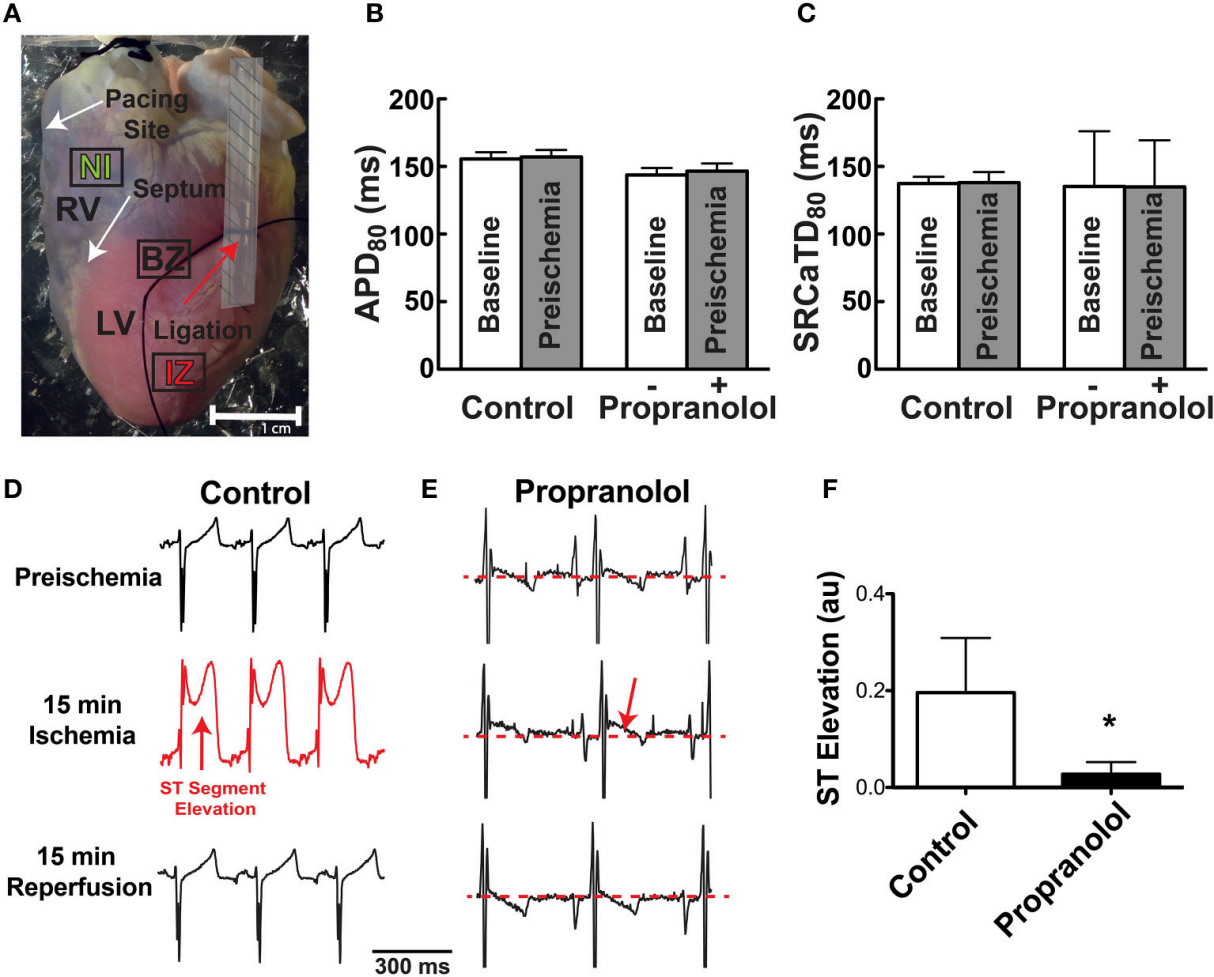
**(A)** Photograph of Langendorff-perfused rabbit heart showing ligation site (red arrow). Following mapping, the ligature was re-tightened and hearts were perfused with Evan's blue dye to demarcate the non-ischemic (NI), ischemic zone (IZ), and border zone (BZ). Dashed area near the ligation site was not included in optical data analysis. **(B,C)** APD_80_ and SR Ca^2+^ transient duration (SRCaTD_80_) at baseline (beginning of experiment) and after 10 min (pre-ischemic time point) to assure stability of the experimental preparation prior to ischemia. Prop-treated hearts received drug (100 nM) immediately following baseline recordings. **(D,E)** Representative ECGs at preischemia, 15 min ischemia, and following 15 min reperfusion in a control **(D)** and Prop-treated heart **(E)**. Prominent ST segment elevation is observed during ischemia in control hearts (red arrow, **D**) and resolves following reperfusion, whereas only minor ST elevation is observed in Prop-treated hearts (red arrow, **E**) despite confirmed perfusion defects with Evan's blue staining. **(F)** Mean ST segment elevation (normalized to QRS amplitude) at 15 min ischemia. Mean±SD. N = 4–5 (*p* < 0.05: ^*^ vs. Control).

Epicardial pacing at a PCL of 300 ms (3.3 Hz) was continuously maintained throughout the experiment except for brief instances at baseline, 15 min ischemia, and 15 min reperfusion, when pacing at PCL = 250 ms was performed to evoke alternans. Baseline electrophysiological parameters were measured at the beginning of the experiment and again 10 min later to assure stability of the experimental preparation. Time = 0 in subsequent plots represents the immediate pre-ischemic time point. The obtuse marginal branch of the LCA was then identified and ligated around a small piece of tubing (Figure 1A). A lead I ECG was continuously monitored and ligation was confirmed by ST segment elevation. Ischemia was maintained for 15 min during which optical files were recorded at 2, 5, 10, and 15 min. The tube within the ligature was then removed to allow for reperfusion and data were again collected at 2, 5, 10, and 15 min of reperfusion. A subset of hearts (*n* = 7) were randomly assigned to acute pre-treatment with the non-specific β-AR blocker, propranolol (Prop, 100 nM). A non-specific β-AR blocker was chosen because of the potentially high levels of local DA and Epi, which may activate β_2_- in addition to the β_1_-ARs activated by high local NE. Prop was added to the perfusate following the first set of baseline measurements and maintained in the perfusate throughout ischemia and reperfusion. The effect of Prop was assessed 10 min after addition and did not significantly impact either V_m_ or SR Ca^2+^ properties (Figures 1B,C, SR Ca^2+^ tau before vs. after Prop: 55.5 ± 3.3 vs. 58.3 ± 3.1 ms, *p* = NS).

Following mapping, the tube was re-inserted through the ligature to reproduce ischemia and Evan's blue dye was injected to demarcate the ischemic region (Figure 1A). This visual demarcation allowed us to define precise areas for analysis that were in the non-ischemic (NI) zone, ischemic zone (IZ), and border zone (BZ: area on the border between NI and IZ). Areas directly adjacent and underneath the tubing were excluded from analysis (Figure 1A, shaded region).

### Data analysis

Relative ST segment elevation on the volume-conducted ECG was quantified as the ratio of the maximal ST amplitude to the QRS amplitude. Optical mapping data were analyzed with two commercially available analysis programs (*BV_Analyze*, Brainvision, Tokyo, Japan; and *Optiq*, Cairn, UK). V_m_ and [Ca^2+^]_SR_ datasets were spatially aligned and processed with a Gaussian spatial filter (radius 3 pixels). For both action potentials (APs) and SR Ca^2+^ transients, activation time was determined at 50% of the maximal (or minimal for SR Ca^2+^) amplitude. APD and SR Ca^2+^ transient durations were calculated at 80% return to baseline. SR Ca^2+^ transient amplitude was defined as systolic–diastolic fluorescence and this value was normalized to the pre-ischemic amplitude since these measurements are uncalibrated and only reflect relative changes in [Ca^2+^]_SR_. SERCA function was assessed using the time constant (τ) of a single exponential fit to the recovery portion of the SR Ca^2+^ trace (from 5 to 90% recovery). The spectral method was used to quantify the magnitude and spatial extent of APD and SR Ca^2+^ alternans as previously described (Wang et al., 2014). For APD, SR Ca^2+^ duration, SR Ca^2+^ amplitude, and SR Ca^2+^ diastolic fluorescence (reflective of uncalibrated relative changes in diastolic [Ca^2+^]_SR_), data were normalized to pre-ischemic (Time = 0) values.

### Statistics

Data are expressed as mean ± standard deviation (SD) and were compared using a two-way ANOVA with Tukey's multiple comparison post-testing. *P* < 0.05 was considered statistically significant. Statistics were performed in GraphPad Prism 7.

## Results

### β-AR inhibition prevents ischemia-induced APD shortening

**Figure 2:**
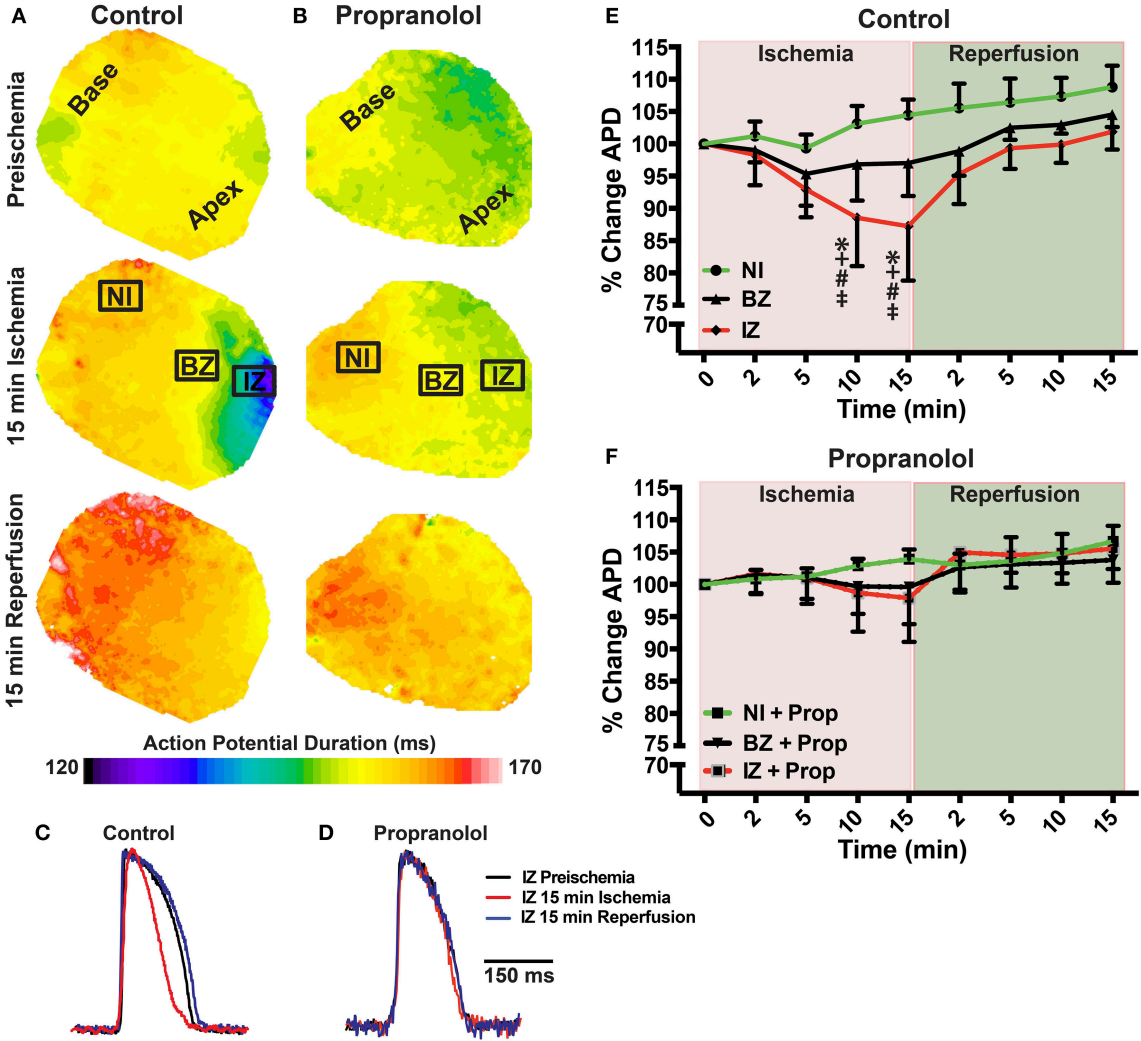
**(A,B)** Action potential duration (APD) maps at preischemia, 15 min ischemia, and 15 min reperfusion in a control **(A)** and Prop-treated heart **(B)**. **(C**,**D)** Example optical action potentials from the ischemic zone (IZ) at preischemia, 15 min ischemia, and 15 min reperfusion. **(E,F)** Summary data of percent change in APD_80_ relative to preischemia (Time = 0 min) for control **(E)** and Prop-treated hearts **(F)**. Mean ± *SD*. *N* = 4–9 [*p* < 0.05: ^*^ vs. NI; + vs. BZ; # vs. preischemia (Time = 0 min); ‡ vs. IZ + Prop].

Prior to assessing the impact of ischemia and β-AR inhibition on electrophysiology and SR Ca^2+^ handling, stability of the experimental preparation was evaluated. Measurements of APD_80_ and SR Ca^2+^ transient duration (SRCaTD_80_) were identical at baseline (beginning of experiment) and 10 min later (pre-ischemic) (Figures 1B,C). To assess the effects of β-AR inhibition on APD shortening during ischemia, maps of APD_80_ were created and data compared from the NI, IZ, and BZ of control and Prop-pretreated hearts. As expected, regional ischemia produced rapid shortening of APD_80_ in the IZ, and to a lesser extent in the BZ, of control hearts (Figures 2A,C,E). There was a gradual, non-significant prolongation of APD_80_ in the NI of control hearts throughout ischemia and reperfusion (Figure 2E). In contrast, Prop pre-treatment led to only minor, non-significant shortening of APD_80_ in the IZ and BZ during ischemia (Figures 2B,D), despite confirmed perfusion defects when Evan's blue dye was injected. There were no statistical differences in APD_80_ observed between the IZ, BZ, and NI zones at any time point in Prop-treated hearts (Figure 2F). Consistent with these findings, ST segment elevation was significantly higher in control vs. Prop pre-treated hearts at 15 min ischemia (Figures 1D–F).

### β-AR inhibition increases SR Ca^2+^ release during early ischemia compared to control ischemic hearts

**Figure 3:**
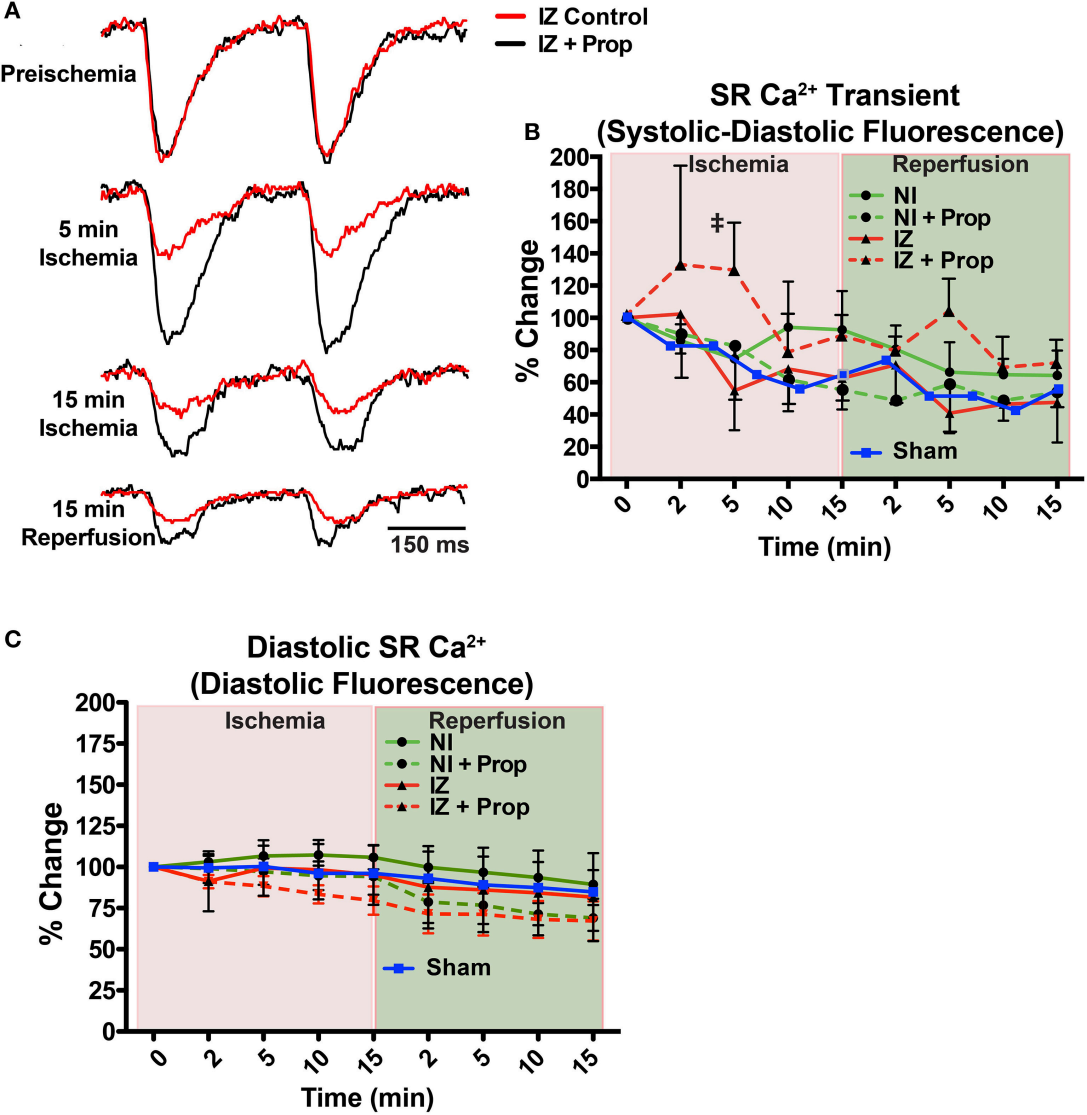
**(A)** Example SR Ca^2+^ traces from the ischemic zone (IZ) during ischemia and reperfusion in a control and Prop-treated heart. **(B)** Summary data of percent change in SR Ca^2+^ transient amplitude (systolic – diastolic fluorescence) relative to preischemia (Time = 0 min) for the non-ischemic (NI) and IZ of control and Prop-treated hearts. A time-controlled sham heart (no ischemia) is shown for comparison. By 5 min ischemia, a significantly larger SR Ca^2+^ transient amplitude is observed in the IZ of Prop-treated hearts vs. IZ of untreated hearts. Mean ± *SD*. *N* = 3–6 (*p* < 0.05: ‡ vs. IZ). **(C)** Summary data of percent change in diastolic SR Ca^2+^ fluorescence (reflective of relative changes in SR Ca^2+^ content) for the NI and IZ of control and Prop-treated hearts as well as a time-controlled sham heart for comparison.

Ischemia induces rapid intracellular acidification, and previous experiments have demonstrated that acidosis reversibly inhibits Ca^2+^ release through ryanodine receptors (RyR) (Xu et al., 1996; Said et al., 2008). To assess the impact of β-AR inhibition on SR Ca^2+^ release, direct optical mapping of free intra-SR Ca^2+^ was performed (Wang et al., 2014, 2015). Relative SR Ca^2+^ release amplitude (measured as systolic–diastolic fluorescence) non-significantly decreased over time throughout ischemia and reperfusion in both the IZ and NI of control hearts and in the NI of Prop-treated hearts (Figure 3). This time-dependent decrease in amplitude is likely due to dye leak and/or extrusion from the SR over time (relative SR Ca^2+^ transient amplitude falls to ~60–70% by 30 min in NI regions, Figure 3B, green lines). In contrast, the SR Ca^2+^ release amplitude in the IZ of Prop-treated hearts tended to increase at 2 min and was significantly larger at 5 min ischemia compared to the IZ of untreated hearts (Figure 3B).

Although previous reports have indicated significant changes in SR Ca^2+^ load throughout ischemia and reperfusion (Valverde et al., 2010), no significant differences in uncalibrated relative SR Ca^2+^ content (measured as diastolic fluorescence) were observed in any region or time point, regardless of treatment (Figure 3C).

### β-AR inhibition prevents ischemia-induced slowing of SR Ca^2+^ reuptake

**Figure 4:**
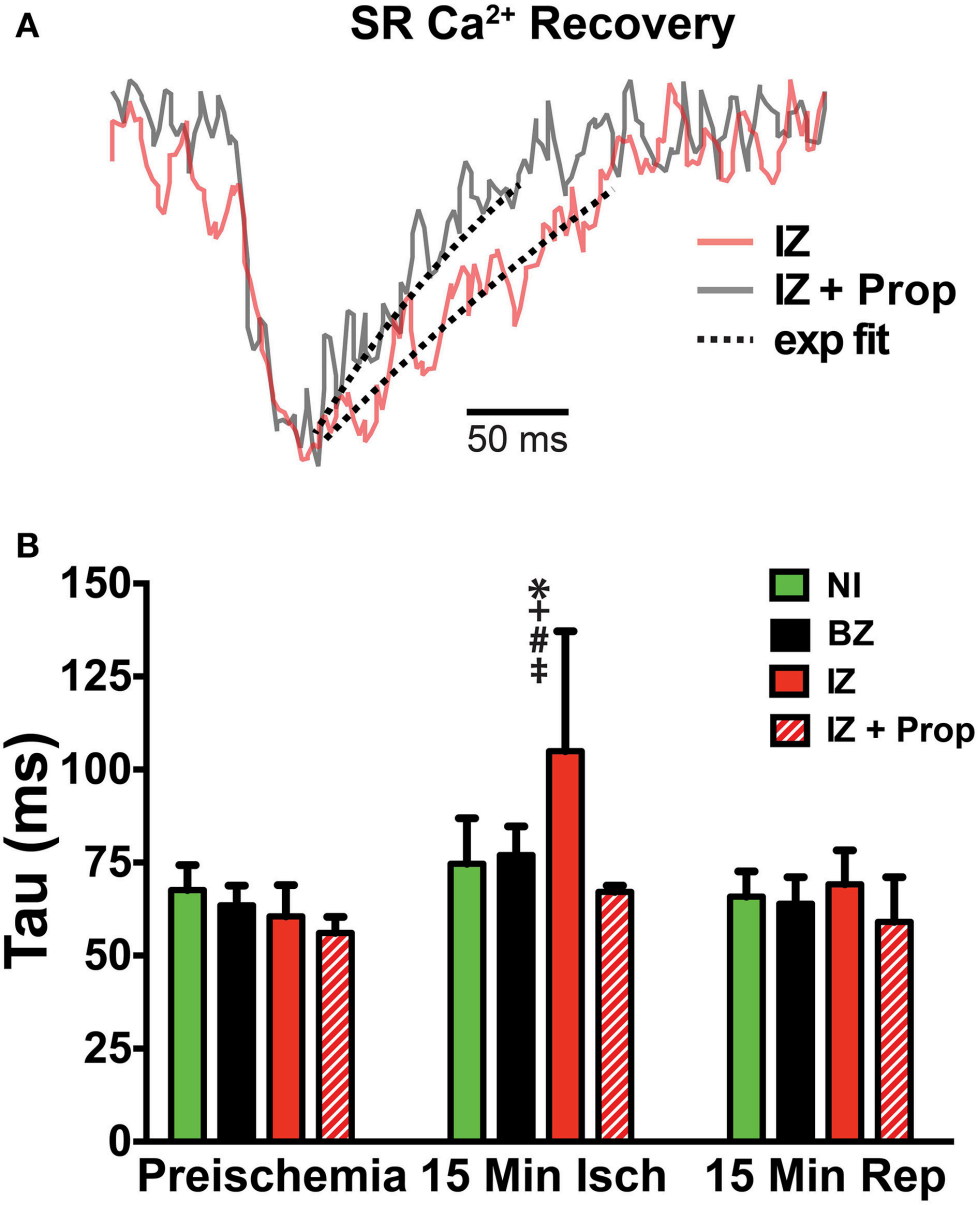
**(A)** SERCA activity was measured as the time constant (*tau*) of a single exponential fit to the recovery portion of the SR Ca^2+^ transient. Example trace from a control heart is in red and a Prop-treated heart is shown in gray. Dashed lines indicate exponential fit. **(B)** *Tau* is significantly increased in the IZ of control hearts at 15 min ischemia, whereas no change in *tau* is observed in Prop-treated hearts at any time point. Mean ± *SD*. *N* = 3–6 [*p* < 0.05: ^*^ vs. NI; + vs. BZ; # vs. preischemia (Time = 0 min); ‡ vs. IZ + Prop].

**Figure 5:**
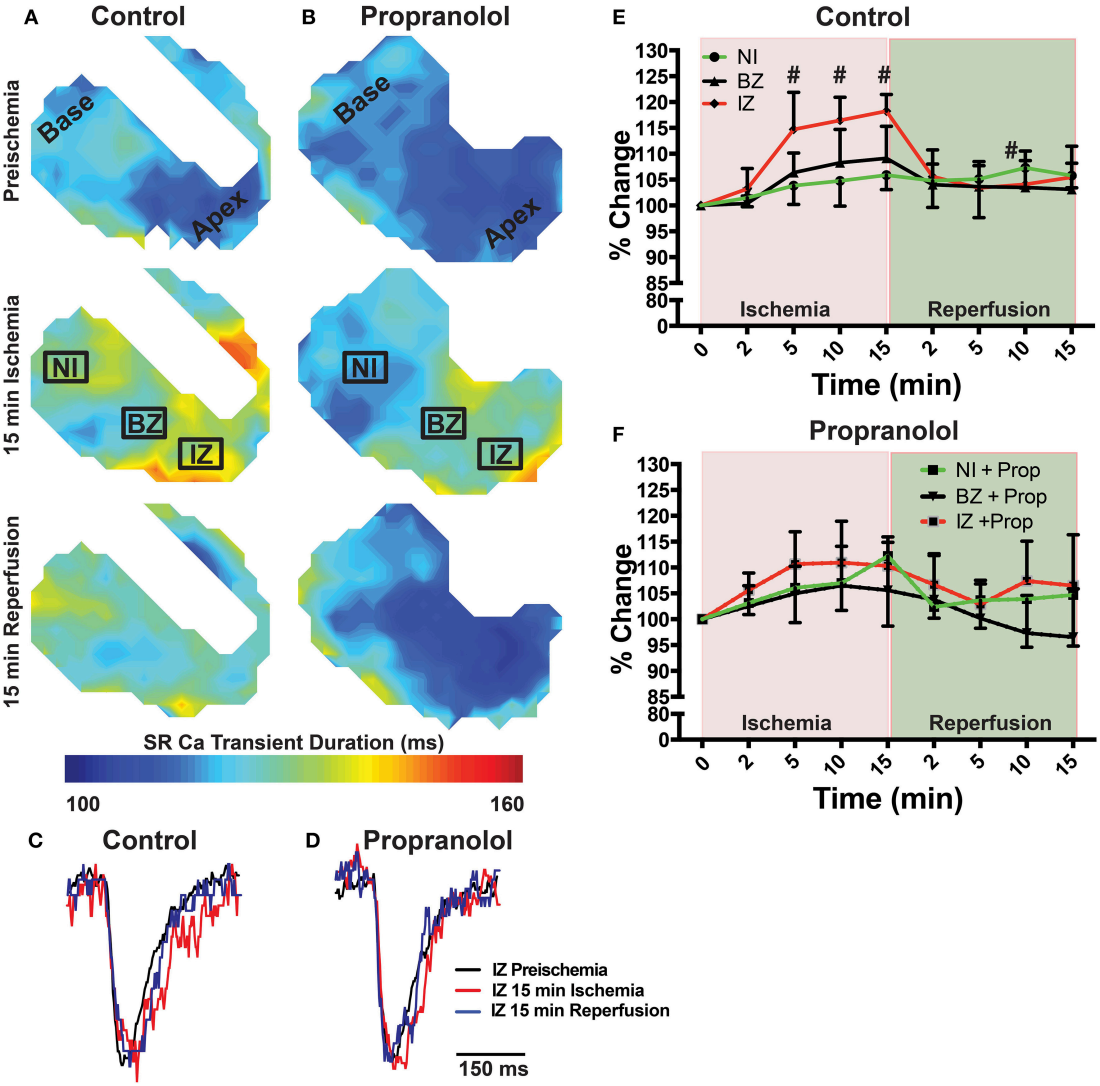
**(A,B)** Maps of SR Ca^2+^ transient duration at 80% recovery at preischemia, 15 min ischemia, and 15 min reperfusion in a control **(A)** and Prop-treated heart **(B). (C,D)** Example SR Ca^2+^ traces from the ischemic zone (IZ) at preischemia, 15 min ischemia, and 15 min reperfusion. **(E,F)** Summary data of percent change in SR Ca^2+^ transient duration relative to preischemia for control **(E)** and Prop-treated hearts **(F)**. Mean ± *SD*. *N* = 3–6 [*p* < 0.05: # vs. preischemia (Time = 0 min)].

Ischemia and associated acidosis are known to inhibit SERCA activity (DeSantiago, 2004). Our data agree with this, as a significant increase in the time constant of SR Ca^2+^ reuptake (*tau*) was observed in the IZ of control hearts, which was reversed upon reperfusion (Figure 4). In non-ischemic conditions, β-AR inhibition may also increase *tau* by inhibiting phosphorylation of phospholamban (PLB). Under ischemic conditions, however, β-AR inhibition prevented the increase in *tau* observed in non-treated hearts (Figure 4). Consistent with these changes in SR Ca^2+^ reuptake, ischemia also led to a significant prolongation of the SR Ca^2+^ transient duration in the IZ of control hearts, but not in Prop-treated hearts (Figure 5).

### β-AR inhibition prevents ischemia-induced APD and SR Ca^2+^ alternans

**Figure 6:**
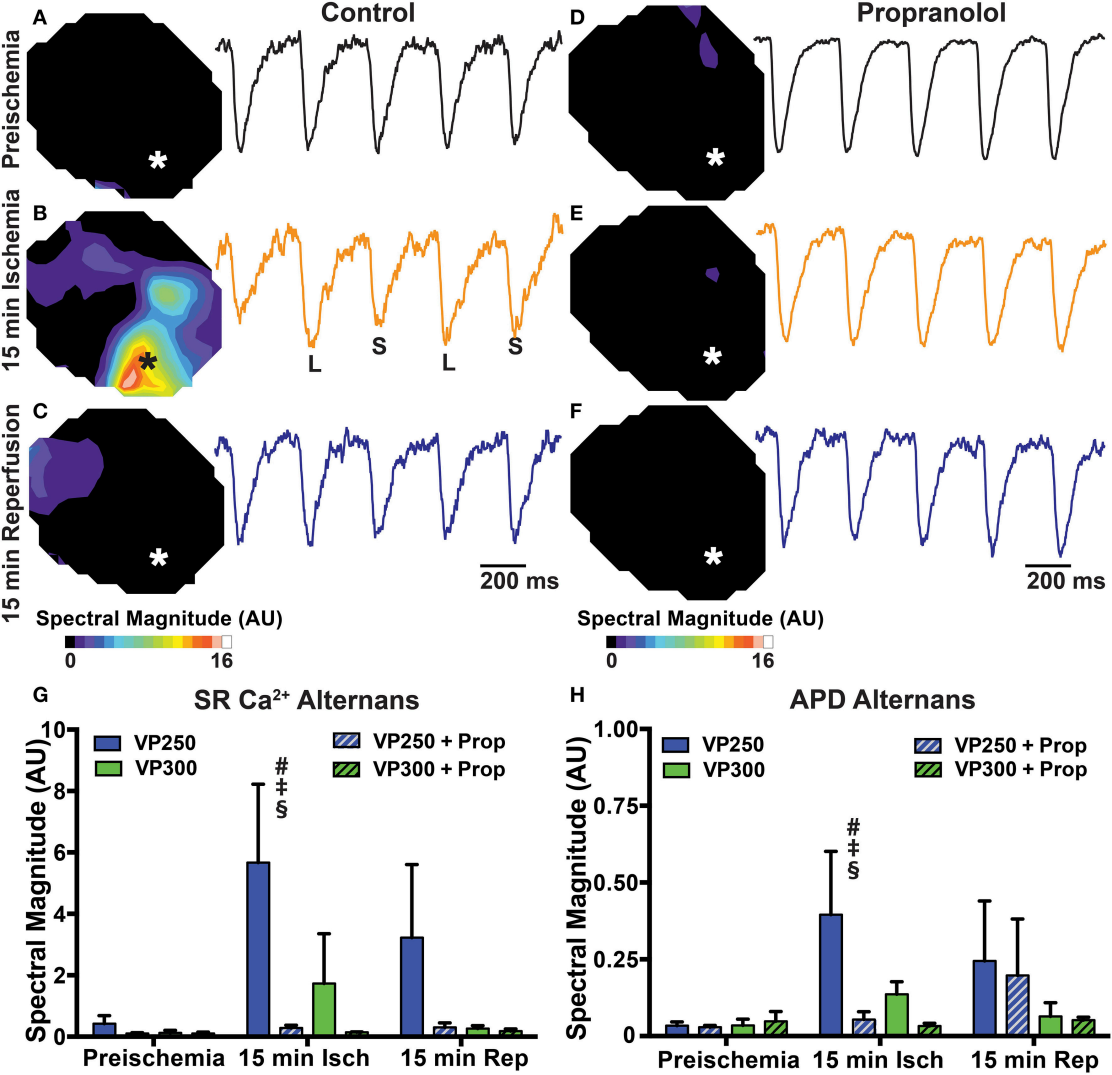
Maps and example traces (from designated areas indicated by ^*^ on the maps) showing SR Ca^2+^ alternans magnitude in a control **(A–C)** and Prop-treated heart **(D–F)** at preischemia, 15 min ischemia, and 15 min reperfusion during constant pacing at a cycle length of 250 ms. Alternans magnitude is increased in the IZ of control hearts at 15 min ischemia but not Prop-treated hearts. Summary data of SR Ca^2+^ alternans magnitude **(G)** and APD alternans magnitude **(H)** at pacing cycle lengths of 300 ms (VP300) and 250 ms (VP250). Mean ± *SD*. *N* = 3–6 [*p* < 0.05: # vs. preischemia (Time = 0 min); ‡ vs. IZ + Prop; § vs. VP300].

Ischemia is known to potentiate arrhythmogenic APD and Ca^2+^ alternans (Qian et al., 2001; Lakireddy et al., 2005). Under non-ischemic conditions, we and others have shown that β-AR stimulation tends to suppress alternans due to acceleration of SR Ca^2+^ release and reuptake (Florea and Blatter, 2012; Wang et al., 2014; Tomek et al., 2017). However, the combined effects of β-AR inhibition and ischemia on APD and SR Ca^2+^ alternans have not been assessed. Alternans was measured at PCLs of 300 and 250 ms at baseline, 15 min ischemia, and following 15 min reperfusion. Neither PCL produced significant alternans at baseline (Figure 6). At 15 min ischemia, however, both APD and SR Ca^2+^ alternans were significantly increased at a PCL of 250 ms in untreated hearts. Prop pre-treatment prevented APD and SR Ca^2+^ alternans at both PCLs during ischemia and reperfusion (Figure 6).

## Discussion

Our results indicate that even when controlling for chronotropic and inotropic effects (with continuous pacing and administration of an excitation-contraction uncoupler, respectively), β-AR inhibition during the acute phase of ischemia prevents APD shortening, alters SR Ca^2+^ release and reuptake kinetics, and prevents arrhythmogenic APD and SR Ca^2+^ alternans. Although some of these findings might be expected based on the known effects of β-AR inhibition and subsequent inhibition of protein kinase A (PKA) activation, other results are novel and suggest a multifaceted role for β-AR inhibition during ischemia.

### Myocyte responses to ischemia

Ischemia results in intracellular acidification and activation of the Na^+^/H^+^ exchanger, leading to an increase in intracellular Na^+^. This Na^+^ overload results in intracellular Ca^2+^ overload via reverse-mode Na^+^-Ca^2+^ exchanger (NCX) activity. At the same time, SR Ca^2+^ release is dramatically reduced due to inhibition of RyR by intracellular acidification and SERCA function is inhibited due to both acidification and a reduction in cellular ATP availability (Rapundalo et al., 1986; Xu et al., 1996; DeSantiago, 2004; Said et al., 2008). Despite inhibition of SR Ca^2+^ release and reuptake, previous studies have shown that intracellular and SR Ca^2+^ levels remain high, but the amplitude and speed of intracellular Ca^2+^ transients are significantly reduced (Valverde et al., 2010) or even eliminated entirely with severe ischemia or anoxia (Stern et al., 1988). Decreases in cellular ATP levels may also lead to the opening of K_ATP_ channels, which are thought to contribute to shortening of the APD during ischemia (Weiss et al., 1992; Aon et al., 2003).

### Neuronal responses to ischemia and local NE release

As in myocytes, cardiac neurons also experience intracellular acidification during ischemia, leading to activation of the Na^+^/H^+^ exchanger and increased intracellular Na^+^. Under non-ischemic conditions, the norepinephrine transporter (NET) is responsible for the reuptake of extracellular NE and DA back into the neuron. NE and DA uptake by NET is coupled to the influx of Na^+^ and Cl^−^ (at a ratio of 1:1:1). During ischemia, NET operates in reverse-mode and transports Na^+^, NE, and DA *out* of the cell. This non-exocytotic release mechanism can increase NE in the extracellular space up to 1000-times the normal plasma NE concentrations within 15 min of ischemia (Schömig et al., 1987; Kurz et al., 1995). Therefore, the myocardial response to ischemia is a combination of hypoxia, acidosis, and elevated adrenergic stimulation.

### Effects of β-AR inhibition during ischemia

If significantly elevated NE results in increased β-AR stimulation during ischemia, one might expect that some of the electrophysiological effects of β-AR signaling would augment or exacerbate myocyte responses to ischemia, while others would oppose the cellular effects of ischemia. For example, during ischemia, the APD shortens primarily due to the opening of K_ATP_ channels (Weiss et al., 1992; Aon et al., 2003). β-AR stimulation also typically results in a shortening of the APD, primarily due to increased I_Ks_. Therefore, ischemia and elevated NE may produce additive effects on APD shortening, and inhibition of β-AR signaling during ischemia would be expected to blunt APD shortening. Indeed, our results are consistent with this interpretation, as APD shortening was significantly diminished when ischemic hearts were pre-treated with Prop (Figure 2). However, Prop pre-treatment not only blunted, but almost completely abolished APD shortening, even at 15 min of ischemia (Figure 2F), suggesting that β-AR inhibition may either be preventing reductions in cellular ATP and subsequent opening of K_ATP_ channels, or inducing ionic currents that are perhaps counterbalancing I_KATP_. Although APD shortening was prevented, refractoriness was not directly measured in the present study and post-repolarization refractoriness occurs during ischemia (Janse et al., 1985; Sutton et al., 2000; Coronel et al., 2012). β-AR inhibition may impact the spatial dispersion of refractoriness and recovery of excitability and these changes likely do not mirror changes in APD. Thus, further studies are needed to directly assess how Prop modifies ischemic post-repolarization refractoriness.

Ischemia reduces the amplitude of SR Ca^2+^ release via inhibition of RyR and slows the rate of SR Ca^2+^ reuptake via reductions in SERCA activity (Rapundalo et al., 1986; Xu et al., 1996; DeSantiago, 2004; Said et al., 2008). β-AR stimulation has the opposite effect, increasing the amplitude of SR Ca^2+^ release and accelerating SR Ca^2+^ reuptake (Bers, 2002). Thus, we hypothesized that β-AR inhibition during ischemia would further slow SR Ca^2+^ cycling kinetics. Contrary to this hypothesis, we observed significant improvements in SR Ca^2+^ handling with β-AR inhibition, including an increase in the amplitude of SR Ca^2+^ release at 5 min ischemia compared to the IZ of untreated hearts (Figure 3), SERCA activity that was similar to non-ischemic conditions (measured as the time constant, *tau*, of SR Ca^2+^ reuptake, Figure 4), and the absence of prolonged SR Ca^2+^ transient duration (Figure 5). The rabbit heart typically displays positive Ca^2+^-V_m_ coupling, meaning that a large Ca^2+^ transient is typically associated with a longer APD (Wang et al., 2014). It is therefore possible that the increased SR Ca^2+^ transient amplitude contributed to the longer APDs observed in Prop-treated ischemic hearts (perhaps countering the effects of I_KATP_). However, due to the bidirectional coupling between Ca^2+^ and V_m_, it is difficult to ascertain the precise contribution of intracellular Ca^2+^ to the observed changes in V_m_ (Shiferaw et al., 2005).

The molecular mechanisms governing the functional improvements in SR Ca^2+^ release and reuptake with β-AR inhibition were not investigated in the present study and are likely multifactorial. Indeed, both SR Ca^2+^ release and reuptake are modulated by several factors, including levels of protein expression and phosphorylation, ATP/ADP, pH, and both intracellular and intra-SR [Ca^2+^] and many of these variables may be impacted by ischemia. The half-life of many Ca^2+^ handling proteins is quite long (e.g., up to 2–3 days for SERCA, 9+ h for PLB) (Andersson et al., 2009; Teng et al., 2015), suggesting that significant changes in protein expression are unlikely within 15 min of ischemia. Indeed, previous studies in the adult rabbit heart indicated that SERCA mRNA levels were decreased following 60 min ischemia, but protein expression remained unchanged (Seehase et al., 2006).

Phosphorylation, on the other hand, is quite dynamic and Vittone et al. (2002) reported maximal PKA phosphorylation of PLB at 20 min ischemia, even though ischemia is known to cause reduced SERCA activity (Rapundalo et al., 1986; Valverde et al., 2010). These results suggest that during ischemia, phosphorylation levels of PLB are no longer a major determinant of SERCA activity and that other factors (e.g., ATP/ADP, pH, [Ca^2+^]) are predominating. Interestingly, Vittone et al. also reported that PLB phosphorylation was reduced with Prop pre-treatment prior to ischemia. SERCA function was not directly assessed in that study, but contractile performance was improved and ischemic contracture was reduced. The authors did not discern the precise mechanisms but speculated that β-AR inhibition may preserve cellular levels of ATP during ischemia (Vittone et al., 2002). Our results are consistent with this interpretation in that Prop pre-treatment likely reduces phosphorylation levels of PLB, yet SERCA function was improved compared to control ischemic conditions (Figures 4, 5), suggesting that other factors, such as ATP availability, may be responsible. Indeed, one of the first steps in the β-AR signaling cascade is the conversion of ATP to cAMP via adenylyl cyclase. Thus, by inhibiting β-AR signaling, significant ATP may be conserved and remain available for other cellular processes during ischemia.

Several previous studies have assessed the impact of modulating β-AR signaling during ischemia. For example, experiments in the isolated mouse heart indicate that spontaneous ventricular arrhythmias are significantly increased when exogenous β-AR agonists (NE and Epi) are added to the perfusate (Stables and Curtis, 2009). Likewise, ischemia-induced arrhythmias can be reduced with prior chemical or surgical sympathectomy (Ebert et al., 1970; Culling et al., 1984), indicating an important role for local nerve-released NE in ischemic arrhythmias. In agreement with the present study, Prop administration immediately following coronary artery ligation in open-chest canine hearts has been shown to prevent APD shortening (Kupersmith et al., 1976) and β-AR blockers have been shown to reduce infarct size, reduce arrhythmias, and improve survival in both ischemic animal models and MI patients (Norris et al., 1984; Hoque et al., 1993; López-Sendón et al., 2004; Dehina et al., 2014). However, none of these previous studies have controlled for chronotropic or inotropic effects; meaning that increasing or decreasing β-AR signaling had direct effects on heart rate and contractility, and therefore, significant impact on energy demand during ischemia.

## Study limitations

Optical mapping of free intra-SR Ca^2+^ with Fluo-5N is not a ratiometric approach. Therefore, the optical signals are uncalibrated and only represent relative changes in SR [Ca^2+^]. Fluo-5N fluorescence decreased over time regardless of ischemic conditions or treatment. This may be due, in part, to dye leak or extrusion from the SR and this time-dependent decrease in signal may obscure more subtle changes in SR Ca^2+^ load during ischemia and reperfusion. Indeed, Valverde et al. (2010) reported a significant increase in SR Ca^2+^ load during ischemia in the mouse heart, whereas no significant differences in relative SR Ca^2+^ diastolic fluorescence (reflective of SR [Ca^2+^] load) were observed under any condition in the present study. Prop is a non-specific β-AR blocker. Therefore, the contribution of β_1_ vs. β_2_ receptor signaling was not assessed in the present study, but this would be an important area for future work. Prop may also have off-target membrane effects that are not fully accounted for and these effects may differ during ischemia. Finally, the present study did not fully dissect the primary effects of Prop during ischemia from the inhibition of excessive β-AR stimulation that may occur (due to local NE release). Experiments in fully denervated or catecholamine-depleted hearts would allow for isolating any potential primary effects of Prop from ischemia-released NE.

## Conclusions

To our knowledge, this is the first study to control for chronotropic and inotropic effects and evaluate the direct impact of β-AR inhibition on action potential and Ca^2+^ handling characteristics during ischemia. Here we report that even when controlling heart rate and contractility, β-AR inhibition during the acute phase of ischemia prevents APD shortening, alters SR Ca^2+^ handling, and prevents arrhythmogenic APD and SR Ca^2+^ alternans. These data are consistent with improved energy metabolism with β-AR inhibition during ischemia. Investigation into the cellular and molecular mechanisms responsible for these effects remains an important area for future study.

## Author contributions

SM, LW, and CR conceived the study, designed experiments, analyzed and interpreted data, and wrote the manuscript. ZW, PD, DL, BH, and RM analyzed and interpreted data and critically revised the manuscript. All authors approved the final version of the manuscript and agree to be accountable for all aspects of the work.

### Conflict of interest statement

The authors declare that the research was conducted in the absence of any commercial or financial relationships that could be construed as a potential conflict of interest.

## Abbreviations

ADP: adenosine diphosphate
APD: action potential duration
ATP: adenosine triphosphate
β-AR: β-adrenergic receptor
BZ: border zone
ECG: electrocardiogram
IZ: ischemic zone
LCA: left circumflex artery
MI: myocardial infarction
NE: norepinephrine
NET: norepinephrine transporter
NI: non-ischemic zone
PCI: percutaneous coronary intervention
PCL: pacing cycle length
Prop: propranolol
RyR: ryanodine receptor
SERCA: sarcoplasmic reticulum Ca^2+^-ATPase
SR: sarcoplasmic reticulum
V_m_: transmembrane potential.
